# Practitioner Review: Differential susceptibility theory: might it help in understanding and treating mental health problems in youth?

**DOI:** 10.1111/jcpp.13801

**Published:** 2023-04-25

**Authors:** Elham Assary, Georgina Krebs, Thalia C. Eley

**Affiliations:** ^1^ MRC Social Genetic and Developmental Psychiatry Research Centre Institute of Psychiatry Psychology and Neuroscience, King's College London London UK; ^2^ Department of Biological and Experimental Psychology, School of Biological and Behavioural Sciences Queen Mary University of London London UK; ^3^ Research Department of Clinical, Educational, and Health Psychology University College London London UK; ^4^ OCD, BDD and Related Disorders Clinic for Young People, South London and Maudsley NHS Foundation Trust London UK

**Keywords:** Resilience, protective factors, life events, gene‐environment interaction, developmental psychopathology

## Abstract

Diathesis‐stress models conceptualise individual differences in propensity for psychopathology as an interaction between environmental risk factors and intra‐individual vulnerabilities. In contrast, the differential susceptibility theory and related frameworks view intra‐individual differences as variations in *sensitivity* to the environments rather than merely *vulnerability* to them. Specifically, they suggest that more sensitive individuals are more affected by the quality of their context, whether positive or negative, than others who are less sensitive. Empirical research over the last two decades has found support for this notion in that greater sensitivity is associated with a greater risk of psychopathology in adverse contexts, but also with lower risk in positive environments. However, despite growing academic and public interest in this field, it is currently unclear to what extent the differential susceptibility model is relevant, or applicable, to clinical practice. The purpose of this review is to focus on the differential susceptibility theory as an alternative explanation of individual differences in mental health and examine its relevance in the treatment of mental health problems in young people. We provide an overview of differential susceptibility and related theories, and current relevant research in the field. We identify potential implications of differential susceptibility models for understanding and treating mental health problems in young people, whilst also highlighting important gaps in research that limit their application at present. Finally, we suggest directions for future research that will assist in the translation of differential susceptibility theories into clinical practice.

## Dedication

Our colleague Dr Rob Keers sadly died on July 5th 2020. Rob was an exceptional person, not just bright and creative but also an incredibly kind and supportive colleague and mentor. One of the main themes of his growing research programme was the exploration of differential susceptibility in young people, and the potential for this approach to inform personalised interventions in the future. The idea to use identical twin differences to explore this was entirely his. The fellowship he was awarded by the Medical Research Council to test this idea resulted in the findings described in Keers et al. (2016). He subsequently gained funding from the Wellcome Trust to further test this hypothesis, work being completed in his absence now. Despite his life and career being cut so short, we feel he is someone who made a significant contribution to the field of child psychology and psychiatry, and we dedicate this review to his memory.

## Background

Empirical research suggests considerable variation in how individuals react both to negative and positive experiences. Whilst chronic and episodic adversity or stress is often associated with the onset of a range of mental health problems, some individuals seem unaffected by these experiences. Similarly, positive influences, such as social support, or therapeutic interventions, do not always elicit the expected positive effects on an individual's well‐being or mental health. These individual differences reflect variations in how sensitive an individual is to the effects of their physical and social environment. Environmental sensitivity has typically been examined within a person × environment (E) interaction design. The person‐level characteristics studied in such interaction design may be broadly categorised as biological (e.g. genes × E), psychological (e.g. temperament × E) or environmental (e.g. poverty × E). Typically, the assumption is made that the studied characteristic renders individuals more vulnerable to the effects of negative environmental factors, in a diathesis‐stress fashion.

**Figure 1:**
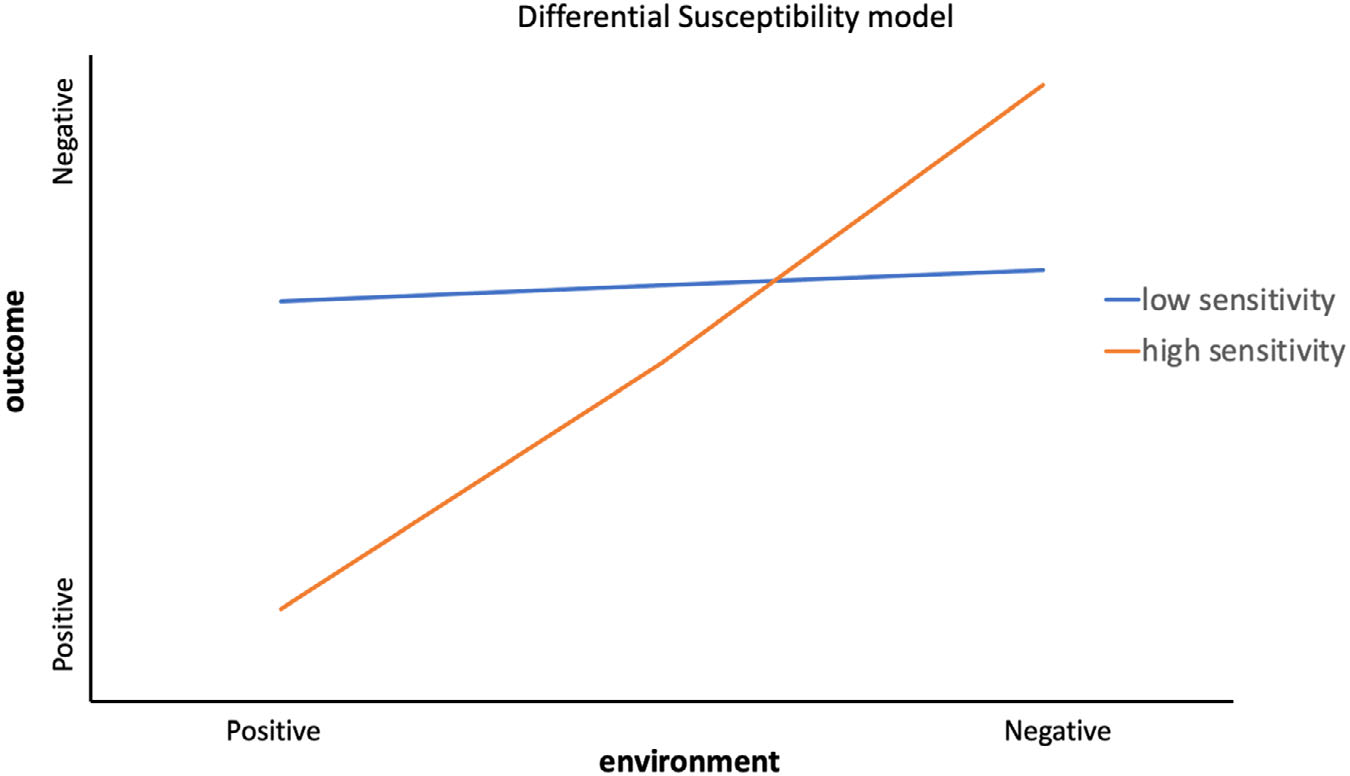
Differential susceptibility model of sensitivity to environmental influences. Higher sensitivity is associated with more negative outcomes in response to negative environmental influences, but also with more positive outcomes in response to positive environmental influences. Figure adapted from Pluess and Belsky (2013). [Color figure can be viewed at wileyonlinelibrary.com]

More recently, it has been proposed that sensitivity to the environment may function in a ‘for better and for worse’ manner (Belsky, Bakermans‐Kranenburg, & van IJzendoorn, 2007). The proponents of this alternative conceptualisation include Sensory Processing Sensitivity (Aron & Aron, 1997), Differential Susceptibility Theory (Belsky et al., 2007; Belsky & Pluess, 2009) and Biological Sensitivity to Context (Boyce & Ellis, 2005; Ellis, Essex, & Boyce, 2005). Though these theories are distinct in various ways, they all suggest that individuals vary in how sensitive/reactive they are towards their physical or social environments, both negative *and* positive. Thus, those who are more sensitive to stressors also respond more to positive influences (see Figure 1). We use the term ‘environmental sensitivity’ from here on, to mean this variation in sensitivity to both positive and negative experiences, and ‘differential susceptibility theories’ as an umbrella term that captures the common underlying promise of the aforementioned theoretical models.

This conceptualisation of environmental sensitivity to include both advantage and disadvantage has considerable implications for our understanding of the aetiology, development, and treatment of mental health disorders. Notably, the personality perspective, which conceptualises the ‘highly sensitive personality’ trait as a measure of environmental sensitivity (e.g. see Slagt, Dubas, van Aken, Ellis, & Deković, 2018), has generated great interest in the non‐academic world. A Google search of the term ‘highly sensitive personality’ returns 250 million hits, as compared to a search for the term ‘neuroticism’ which returns 64 million hits. This includes many online platforms claiming to be able to quantify one's own or one's children's sensitivity using interviews, questionnaires or DNA (e.g. GenomeLink). They promote a wealth of lifestyle, wellness, clinical and psychotherapeutic services to ‘make the most of your sensitivities’, or similar. This is a worrying trend, as despite increasing public and research interest, there are important gaps in literature including (a) how to most appropriately index individual differences in sensitivity, and its stability and change across life span, (b) the underlying mechanisms and (c) how sensitivity is related to mental well‐being, and the development, maintenance and treatment of mental health problems. The purpose of this review is to consider current differential susceptibility research and examine whether, and how, it might inform clinical practice, particularly with children and adolescents. We will also suggest future directions for research that can facilitate the translational potential of differential susceptibility theories.

## Differential susceptibility theory and related frameworks

In this section, we briefly review three different but conceptually related theories that use a differential susceptibility framework to explain individual differences in environmental sensitivity.

### Differential susceptibility theory

This is an evolutionary‐inspired developmental model that considers potential disadvantages as well as advantages of individual differences in environmental sensitivity by examining effects on inclusive fitness (Belsky et al., 2007; Belsky & Pluess, 2009). This theory suggests that natural selection would favour both high and low‐sensitivity types. Specifically, whilst low susceptibility may predict resilience in the face of adversity, and, therefore, reproductive fitness, higher susceptibility could also lead to increased reproductive fitness through enhanced adaptation to positive environments. Initial support for this theory was drawn from the developmental psychology literature, particularly that relating to parenting studies, and later from gene–environment interaction studies (Belsky et al., 2007; Belsky & Pluess, 2009, 2013). A related concept, that emphasises a greater propensity to benefit from positive influences has seen been proposed.

### Biological sensitivity to context

This theory focuses specifically on physiological differences in reactivity such as arterial pressure, cortisol production or immune reactivity, to environmental stimuli (Boyce & Ellis, 2005; Ellis et al., 2005). Sensitivity is defined as neurobiological susceptibility to cost‐inflicting as well as benefit‐conferring environments and operationalised as an endophenotype reflecting heightened reactivity in one or more stress response systems. More reactive physiological reactivity systems are proposed to increase susceptibility to negative environments, but also to resources and support (e.g. cooperative information, social opportunities). This model emphasises the role of early environments in shaping these physiological differences in sensitivity to environmental stimuli, based on the evolutionary notion of conditional adaptation. As such, high sensitivity is thought to develop mainly in response to both extreme negative and positive environments (Ellis & Boyce, 2011; Ellis, Jackson, & Boyce, 2006; Ellis, Oldehinkel, & Nederhof, 2017).

### Sensory processing sensitivity theory

This theory views environmental sensitivity as a common, stable personality trait, that is, evidenced in children and adults alike. Highly sensitive personality is thought to be marked by greater depth and breadth of processing of emotional and psychological stimuli, lower threshold of reactivity to stimulation, greater attention to and awareness of aesthetics and subtleties in the environment, and behavioural inhibition when faced with novel stimuli (Aron & Aron, 1997). Built on Jung's theory of innate sensitiveness, it is suggested that the thorough processing of environmental stimuli in highly sensitive individuals enables their detection of subtilties, whether distressing or positive (Aron, 2004). Sensitivity is not considered a disorder, rather it is a type of personality trait. However, sensitive persons may be at higher risk of developing mental health problems when exposed to stressors than less‐sensitive individuals. In the absence of stressors, highly sensitive people would not be at elevated risk of these difficulties and may even be at lower risk since they are also more attuned to supportive cues.

### Proposed mechanisms

The exact mechanisms underlying environmental sensitivity are currently unknown, though all three of these theories have suggested potential biological mechanisms. The differential susceptibility theory has mainly emphasised the involvement of dopaminergic and serotoninergic circuitry, that is, implicated in responsivity to reward and punishment, and amygdala reactivity as one of the several central nervous system mechanisms (Belsky & Pluess, 2009). Variations in these systems are suggested to relate to reward threshold, differences in attention, orientation of response, response regulation and emotional reactivity, all‐important domains in the extent of responsivity/reactivity to environmental stimuli. Biological sensitivity to context has emphasised the role of stress response systems such as autonomic, adrenocortical or immune reactivity in response to psychosocial stressors (Boyce & Ellis, 2005; Ellis et al., 2005). Variations in such psychobiological reactivity are thought to reflect individual differences in environmental sensitivity. Finally, the sensory processing sensitivity theory suggests that brain regions/processes involved in awareness of and attention to subtle stimuli, emotional responsivity, empathy to others' affective cues and depth of processing of the stimuli best capture the underlying mechanism of heightened environmental sensitivity (Aron & Aron, 1997).

There is modest but growing evidence to support the involvement of the various hypothesised systems. In studies with children, high salivary cortisol levels have been associated with more maladaptive socio‐cognitive outcomes in the context of high adversity, but also with better outcomes in the context of low adversity (Obradovic, Bush, Stamperdahl, Adler, & Boyce, 2010). Other studies with adults have found that highly sensitive individuals showed greater activation in regions of the brain involved in attention and action planning, awareness, integration of sensory information and empathy, whilst viewing photos of their romantic partners and of strangers displaying positive, negative or neutral facial expressions (Acevedo et al., 2014).

## Measuring individual differences in environmental sensitivity

There are three main approaches to studying environmental sensitivity from a differential susceptibility perspective. In the most widely used approach, a biological or psychological marker thought to reflect individual differences in environmental sensitivity (e.g. amygdala reactivity/difficult temperament), is examined in an interaction design. If the candidate sensitivity marker moderates the outcome of the environmental exposure (e.g. parenting) ‘for worse’ at the negative end of the environmental spectrum, but also ‘for better’ at the positive end, then the marker is considered to capture environmental sensitivity. This approach has been instrumental in showing that many markers previously considered as vulnerability factors, instead reflect a differential susceptibility model.

A second approach considers individual differences in environmental sensitivity from a personality perspective, holding that such variations are stable tendencies that can be characterised via self‐report questionnaires (e.g. Highly Sensitive Person; Aron & Aron, 1997) or observational measures (e.g. Highly Sensitive Child‐Rating System; Lionetti, Aron, Aron, Klein, & Pluess, 2019). This approach offers a quantitative, phenotypic measure of individual differences in environmental sensitivity, and facilitates exploring its associations with a range of clinical and health outcomes.

A third approach indexes individual differences in environmental sensitivity by using genetic data and twin samples (Keers et al., 2016). Because monozygotic (MZ) twin pairs are genetically identical, any phenotypic differences between members of an MZ twin pair must reflect non‐shared (i.e. child‐specific) environmental influences, alongside measurement error. These can include events that only member of the pair experiences, as well as different responses to the same event. This latter element thus captures differential responsivity to the environment. This method becomes even more informative when genome‐wide genetic data is available. In a population of MZ twins, those pairs who carry genetic variants that increase their sensitivity to the environment would thus show greater intra‐pair phenotypic differences. Notably, these phenotypic differences reflect the total effects of *all* non‐shared environmental factors, both positive and negative. A genome‐wide association study (GWAS) of MZ twin differences can therefore estimate the extent of associations between genetic variants and environmental sensitivity. Using the estimates (beta‐coefficients) from this initial GWAS, it is then possible to obtain a personalised genetic index of environmental sensitivity by creating polygenic scores constructed from the cumulative effect of the genetic variants across the genome.

In the next section, we will review the clinically relevant research studies that have used these three main approaches to investigate environmental sensitivity and its associations with mental health. It is important to note that whilst we present evidence from these different approaches together, to our knowledge no studies to date have attempted to compare or combine these different methods. It is, therefore, unclear whether they are tapping into the same or different constructs of environmental sensitivity. Therefore, we must be cautious when interpreting the findings from one phenotype/marker of sensitivity to support findings of, or make inferences about the function of, other sensitivity markers.

## Differential susceptibility research findings

In this section, we review three areas of research examining the role of environmental sensitivity within the differential susceptibility framework on different types of outcomes. First, are studies exploring developmental outcomes in response to early childhood experiences. Second is research examining mental health outcomes. Third, we end with intervention studies assessing psychological treatment outcomes.

### Environmental sensitivity and developmental outcomes

As noted previously, the earliest support of the differential susceptibility theory came from parenting studies (Belsky & Pluess, 2013). Recent research has built on earlier findings, showing that various indices reflecting environmental sensitivity, moderate the developmental outcomes of parenting and psychosocial environmental factors for better and for worse. For example, a review of different child characteristics showed that children with a more difficult temperament (marked by being difficult to sooth and crying easily), were more vulnerable to negative parenting, but also profited more from positive parenting, supporting the differential susceptibility model (Slagt, Dubas, Dekovic, & van Aken, 2016). A study of 264 children (mean age 4.7), found that those children scoring higher on the highly sensitive questionnaire were more responsive to changes in parenting behaviour in both positive and negative directions than those with lower scores (Slagt et al., 2018). Specifically, highly sensitive children displayed increased externalising problems in the context of negative parenting but decreased externalising problems in the presence of positive parenting, over a 2‐year period. Other studies have examined the interaction between sensitivity and parenting style on mental health outcomes in preschool‐aged children (Lionetti et al., 2019, 2021). For example, sensitivity, measured via the observational rating scale (HSC‐RS), was found to moderate the association between permissive parenting and externalising problems at age 3 (Lionetti et al., 2019). Sensitivity also moderated the association between authoritative parenting style and social competence at ages 3 and 6 years.

Two recent studies used physiological markers to test the differential susceptibility hypothesis in adolescent girls. In the first study, the link between parent–child relationship quality and depressive symptoms and neuronal activity (their marker of sensitivity) was examined for 45 adolescent girls who were exposed to social exclusion during an fMRI scan (Rudolph et al., 2020). Stressful parent–child relationships predicted depressive symptoms in girls with high and moderate, but not low, dorsal anterior cingulate cortex, subgenual anterior cingulate cortex, and anterior insula activation, during exclusion. However, in the context of supportive parent–child relationships, neural activation to exclusion predicted especially low levels of depressive symptoms, supporting a differential susceptibility to the environment model. In the second study, they examined whether rejection sensitivity was related to amygdala‐rVLPFC connectivity (which has been linked to poor emotion regulation), in the context of victimisation (Rudolph et al., 2021). Adolescent girls with high (but not low) rejection sensitivity and a history of peer victimisation showed less‐effective neural regulation of emotion (i.e. more positive amygdala‐rVLPFC connectivity). Consistent with a differential susceptibility model, adolescent girls with high rejection sensitivity but low peer victimisation, showed particularly effective neural regulation of emotion (i.e. more negative amygdala‐rVLPFC connectivity). They found similar results when using a behavioural index of emotion regulation, supporting a differential susceptibility pattern of interaction.

Using the MZ twin differences approach described earlier, one study examined how a polygenic score of sensitivity moderated the effects of parenting on children's emotional problems (Keers et al., 2016). They found that for children with a high genetic sensitivity score, negative parenting was associated with higher emotional problems and positive parenting with lower emotional problems. In contrast, for children with a low genetic sensitivity score, parenting had little effect on emotional problems.

Most of the studies reviewed thus far have measured sensitivity only at one time point. To our knowledge, there have been no longitudinal or life‐course analyses of environmental sensitivity. It is, therefore, unclear how sensitivity changes across the life span. Specifically, do individuals who are deemed to be more sensitive in childhood continue to be so in their adulthood and older age? If so, does sensitivity function in the same ‘for better and for worse’ manner across the lifespan, or does childhood sensitivity change in response to cumulative life experiences? The latter would reflect a more dynamic view of sensitivity, which would impact how we consider sensitivity and its effects on mental health across the lifespan. This is an important gap in research that merits further investigation.

### Environmental sensitivity and psychopathology

In studies with adults and adolescents, a highly sensitive personality has been associated with increased levels of numerous psychopathology symptoms and poor mental health outcomes. These include anxiety and depression symptoms (Bakker & Moulding, 2012; Liss, Mailloux, & Erchull, 2008; Liss, Timmel, Baxley, & Killingsworth, 2005; Meredith, Bailey, Strong, & Rappel, 2016; Yano & Oishi, 2018), autism symptoms and alexithymia (Jakobson & Rigby, 2021; Liss et al., 2008), and emotional regulation problems (Brindle, Moulding, Bakker, & Nedeljkovic, 2015). Furthermore, higher sensitivity has been associated with lower levels of life satisfaction, extraversion, and higher levels of neuroticism (Booth, Standage, & Fox, 2015; Pluess et al., 2018; Smolewska, McCabe, & Woody, 2006; Sobocko & Zelenski, 2015). Highly sensitive adults have also been found to report poorer physical ill health (Benham, 2006), greater displeasure with work and requiring longer psychological recovery times (Anttila et al., 2018; Evers, Rasche, & Schabracq, 2008), burn out syndrome (Golonka & Gulla, 2021), greater COVID‐19 pandemic stress and internalising problems (Burgard, Liber, Geurts, & Koning, 2022; Iimura, 2022), and higher levels of nightmare distress in the context of poor mental health (Carr, Matthews, Williams, & Blagrove, 2021).

As with the developmental outcomes, much of this literature also involved cross‐sectional research, and is correlational. Given this, the potential *causal* role of sensitivity in the development of these disorders is unclear, despite theoretical contentions. It is possible individuals become more sensitive to their environment as a consequence of mental health problems, and/or that the observed cross‐sectional correlations between sensitivity and symptoms reflect shared aetiology. Indeed, a recent study found the covariations in sensitivity, neuroticism and low extraversion were due to shared genetic influences on these traits (Assary, Zavos, Krapohl, Keers, & Pluess, 2021).

Despite lack of knowledge on the exact mechanisms underlying these associations, research findings on environmental sensitivity seem to suggest a greater burden of risk for psychopathology in highly sensitive individuals. This may be due to the limitations of the current personality measures of sensitivity, with an over‐representation of negative sensitivity items in the questionnaires and under‐representation of the positive ones. Furthermore, the absence of associations with more positive outcomes for highly sensitive persons reflects an overemphasis in the literature on studies of psychopathology and the scarcity of research that has included positive influences and outcomes. The main body of evidence in support of the ‘for better’ associations with higher sensitivity consist of treatment studies which are reviewed next.

### Environmental sensitivity and response to therapeutic interventions

Though rather scarce, studies using various indices of sensitivity have found support for the notion that highly sensitive individuals benefit more from therapeutic interventions. For example, one study examined the association between sensitivity and change in depressive symptoms following a school‐based resilience‐promoting program in 363 adolescent girls (Pluess & Boniwell, 2015). They found that adolescents with highly sensitive personality scores showed significantly lower depression scores at 12 months follow‐up, whereas those with less sensitive personalities did not. Another study examined the effects of sensitivity on reducing victimisation, internalising and externalising symptoms following a randomised control trial school‐based anti‐bullying intervention (*N* ~ 2000; Nocentini, Menesini, & Pluess, 2018). As expected, pupils who received the intervention showed lower symptoms compared to controls post‐treatment. Importantly, outcomes of those in the intervention group varied by sex and highly sensitive child scores. Specifically, highly sensitive boys showed significantly larger reductions in victimisation and internalising symptoms following the intervention, than less sensitive boys. Sensitivity did not moderate outcomes for girls. For externalising problems, there was no significant effect of sensitivity in either girls or boys.

The association between sensitivity and therapy outcomes has also been examined using genetic designs. Early genetic studies of differential susceptibility examined sensitivity exclusively using the candidate gene approach, usually selecting serotonin and dopamine‐related genetic variants as sensitivity markers. Whilst meta‐analyses suggest consistency with differential susceptibility theories (Bakermans‐Kranenburg & van IJzendoorn, 2011; van IJzendoorn, Belsky, & Bakermans‐Kranenburg, 2012), we will not review these studies here for two reasons. First, they have been extensively reviewed elsewhere (e.g Belsky & Pluess, 2013). Secondly, despite initial enthusiasm for this approach in the field of psychiatric genetics, and in environmental sensitivity research, the candidate gene method is now considered both flawed and redundant, largely due to discounting of the complex genetic architecture of psychological traits and low replicability of findings (Duncan & Keller, 2011).

Reflecting the move towards genome‐wide approaches, a more recent study used a genome‐wide polygenic score of sensitivity to examine response to psychological treatment delivery mode for child anxiety disorders (Keers et al., 2016). Using the MZ differences approach to construct the polygenic score, they found that the genetic score of sensitivity moderated response to the mode of delivery of cognitive behavioural therapy (CBT). Specifically, children with high genetic sensitivity scores showed greatest improvement in symptoms if they received individual CBT, moderate improvements for group CBT, and least improvement if they received a brief parent‐led CBT. In contrast, those with a low genetic score of sensitivity responded similarly regardless of treatment delivery type. These effects are potentially clinically meaningful, with children with high genetic sensitivity scores achieving remission rates of 70.9%, 55.1% and 40.6% for individual, group and brief parent‐led CBT, respectively. These results suggest that whilst more sensitive children benefited more from high‐intensity therapy delivery, less sensitive children did equally well with all three types of treatment delivery.

Overall, the findings could indicate that higher sensitivity may be associated with higher risk of developing mental health problems in the context of psychosocial adversity but also with deriving benefits from more individualised therapeutic interventions. We must note that the findings are modest and require further replication. Additionally, we highlight that most studies for treatment response in this field, and those reviewed herein, have been conducted in children. It, therefore, remains an empirical question as to whether the same patterns would emerge for adults.

## Differential susceptibility theory and its potential impact on clinical practice

The diathesis‐stress perspective is currently the dominant person‐by‐environment interaction model of individual differences in mental health, with perhaps less awareness of alternative models such as differential susceptibility. Greater awareness of the theoretical propositions and research findings in this field could be influential in how psychopathology is viewed and treated by practitioners. We must emphasise that, at present, this knowledge, whilst exciting, cannot yet be used to inform direct clinical practice. Based on current research, and conditional on further evidence, the next section considers three main areas where the differential susceptibility approach could potentially influence clinical practice.

### Psychoeducation to highlight *sensitivity,* not just vulnerability

At the heart of the differential susceptibility theory is the idea of sensitivity to positives as well as negatives, rather than the vulnerability narrative of mental health. Awareness of the research on sensitivity would be an important step in reducing the stigma of mental health disorders such as depression as a function of inherent vulnerability factors. The knowledge that it may be greater sensitivity, rather than greater vulnerability, that influences symptoms could provide a more positive way of understanding mental health problems and bring relief to patients and parents attending Child and Adolescent Mental Health Services (CAMHS).

At the assessment stage, clinicians could consider whether high sensitivity may be a useful component of a young person's formulation. When relevant, subsequent psychoeducation with patients and parents could emphasise that whilst greater sensitivity to stressors may be *one* risk factor for mental health problems, it does not mean that the development and persistence of mental health problems are *inevitable*. This is because the onset of psychiatric disorders, such as depression, depends on the complex interaction between their existing positive and negative environmental contexts. Therefore, a higher load of positive/protective factors could reduce the impact of the negative events. From this perspective, building resilience and creating a more supportive environment, ensures better chances of recovery and success following significant stressful life events for such individuals. Furthermore, psychoeducation could emphasise the ‘for better’ aspect of being highly sensitive. Parents of highly sensitive children may consider their child to have a ‘difficult’ temperament and feel relatively hopeless that they can instigate change. Reframing the child as being highly sensitive during the formulation and psychoeducation phase of treatment will enable the parent to consider the possibility that their child may be more responsive, not less responsive, to psychological and systemic interventions, including changes in parenting practices. This message is likely to be empowering and motivating both for parents and young people themselves, which may in turn promote engagement with therapeutic interventions. In this vein, therapeutic optimism and treatment expectancy has been shown to be associated with better compliance and treatment outcomes (Curry et al., 2006; Westra, Dozois, & Marcus, 2007). Research findings that sensitive individuals show greater reduction in their symptoms following therapeutic interventions, can also serve as an important motivating factor in encouraging treatment continuity. The possibility of more positive outcomes for the client and therapist, and also for parents undergoing positive parenting interventions, can be drawn on to motivate service users to continue with the intended course of treatment, despite challenges.

### Considering sensitivity in treatment

The findings regarding responses to intervention are modest, but they indicate that outcomes may differ for highly sensitive individuals as a function of intervention characteristics such as delivery mode. As summarised above, one study has shown that more sensitive individuals respond better to individual CBT, whilst less sensitive individuals benefit equally from group or individual CBT (Keers et al., 2016). Should these findings be replicated and extended into other forms of treatment, they may help to inform clinical decision‐making. For example, findings may suggest that clinicians could helpfully prioritise highly sensitive individuals for individual as opposed to group treatment. At the very least, we suggest that environmental sensitivity merits consideration as a contender along with other relevant variables to maximise prognostic value and inform clinical decision‐making when drawing up personalised treatment plans.

Therapeutic outcomes amongst highly sensitive persons could theoretically be optimised using two broad approaches. First, interventions could aim to *modify environmental factors* and reduce stressors. This could be possible through systemic approaches that are aimed at enhancing the environmental contexts for those who are highly sensitive, in order to improve their mental health outcomes. We acknowledge that some systemic approaches aimed at improving environmental variables are hard to implement, for example, those involving macro environments such as school culture. It is however possible for practitioners to identify the aspects of the environment that can be modified and support the individual in navigating these changes. For example, involving parents and other family members in treatment may provide an opportunity to optimise the home environment. Of note, family‐based approaches are standard for some childhood mental health disorders (e.g. externalising disorders), but for emotional disorders (e.g. anxiety and depression) adolescents are often predominantly seen alone. This may not be optimal, given that anxiety and depression are often associated with critical and overprotective parenting styles and other types of family dysfunction (Lebowitz et al., 2013; McLeod, Weisz, & Wood, 2007). For highly sensitive individuals in particular, interventions that address these wider family factors could have a beneficial effect.

Second, interventions could aim to *increase resilience* amongst those who are highly sensitive, in order to better equip them to deal with environmental stressors. Highly sensitive young people may be prone to experiencing more frequent or intense emotional responses to environmental stimuli, relative to less sensitive individuals. Therefore, it may be beneficial to help highly sensitive individuals to understand that they have this tendency, for better and for worse, and to develop strategies to regulate their emotional reactivity. A wide range of techniques could be utilised for this purpose, depending on the young person's individual formulation (including presenting difficulties and developmental level) (Moltrecht, Deighton, Patalay, & Edbrooke‐Childs, 2021). For example, cognitive techniques could be used to target relevant negative appraisals (e.g. ‘I can't cope’). Problem‐solving skills could be used to help the highly sensitive young person tackle stressful situations in a productive way, in order to reduce distress. Similarly, mindfulness and acceptance techniques could be used to encourage non‐judgmental acceptance of emotions, thereby reducing escalation of negative emotions. For some highly sensitive young people, a combination of these approaches may be optimal. For example, it may be helpful to support the young person in learning to distinguish between the negative feelings and situations that they should respond to (e.g. using problem‐solving), and those that they should strive to accept. This could assist in developing a more objective perspective on emotional state, enhance self‐control and lower emotional reactivity.

There has been little research on the efficacy of these specific therapeutic strategies for promoting resilience in highly sensitive young people. However, given research shows that acceptance of negative affective states partially mediated the association between sensitivity and symptoms of depression (Brindle et al., 2015), and that associations between sensitivity and anxiety were only found when mindfulness and acceptance were low (Bakker & Moulding, 2012), mindfulness‐based therapies may be helpful. A qualitative study of highly sensitive individuals also found that they considered positivity, acceptance, and reflection as helpful psychological strategies for coping with negative thoughts (Bas et al., 2021). Also, a recent study that used a computational‐based approach indicated that highly sensitive individuals internalise emotional regulation strategies modelled by another (not highly sensitive) person (Tran, Treur, & Tuinhof, 2018). The results highlight the potential benefit of therapists practising implementing emotion regulation strategies with highly sensitive young people *within the therapy sessions*, as opposed to relying solely on the young practising them in between sessions.

### Considering sensitivity in relapse prevention

Relapse prevention is an important component of most psychological treatment protocols but may be particularly important for highly sensitive young people. Environmental stressors are a common trigger for relapse across multiple disorders. Since highly sensitive children are more reactive to their environment than less sensitive children, it follows that they may be at especially high risk of relapse if they experience future stressors, although this is yet to be empirically tested. Nevertheless, clinicians might helpfully anticipate a risk of relapse in response to stressful life events or transitions such as school examinations, moving house, parental divorce, and bereavement. Ensuring a robust relapse prevention plan for young people that is shared with parents, might help to mitigate the risk of symptom recurrence. This could include spotting ‘early warning’ signs and encouraging early help‐seeking. There may also be a benefit to services offering more frequent follow‐up appointments and longer term follow‐up appointments to highly sensitive young people, and/or rapid access to booster sessions at times of stress. In the absence of research evidence, the effectiveness of these proposed approaches is speculative. Future studies are needed to examine whether, and if so, why, highly sensitive individuals may be at higher risk of relapse.

## Challenges in applying theory to clinical practice

Whilst in the previous section, we have identified several ways in which differential susceptibility could potentially impact clinical practice, there are challenges in applying these research findings in a meaningful way. One of the main challenges is the absence of clinically meaningful cut‐offs. Whilst there have been suggestions as to the cut‐off points that constitute ‘high sensitivity’ on the child measure (Pluess et al., 2018), it remains unclear how sensitivity relates to clinically diagnosed mental health problems. This is important because current research has almost exclusively been conducted with self‐report symptom measures and not clinically diagnosed samples. Relatedly, the most widely used self‐report questionnaire measures (HSP and HSC) have been criticised for being biased towards the negative aspects of sensitivity, with a greater number of items in the questionnaire capturing unpleasant/negative responses to one's context (see Greven et al., 2019). This is an important limitation because an overemphasis on the negative could lead to underestimating potential associations with positive outcomes including well‐being.

The polygenic score method of sensitivity is promising in offering a more objective quantitative measure of sensitivity, but it currently predicts a very small proportion of the variance in sensitivity. Work to build this predictive power depends on ascertainment of large enough samples to considerably improve statistical power for detecting genetic effects. Physiological measures are valuable in experimental research but are less practical and harder to implement in clinical practice. As such, measures of sensitivity, including questionnaires, have limited clinical utility at present. The question of how best to index environmental sensitivity is, therefore, an important one that is yet to be settled. It is paramount that further research is undertaken in this area if this phenotype is to fulfil its potential as a clinically useful variable in provision and treatment of mental health disorders.

The second challenge is how to improve on formulating appropriate treatments for sensitive individuals who develop mental health problems when little is known about the underlying processes involved. Understanding the mechanisms that confer both disadvantages and advantages is crucial in developing targeted strategies for prevention and treatment of mental health disorders. As such, further research is required to understand the psychobiological processes that enable these contrasting outcomes, for example, through prospective longitudinal studies. Several other important questions remain, including whether sensitive individuals who show enhanced response to specific types of intervention have a different profile of sensitivity compared to those who are more sensitive to the negative aspects of adversity. Vantage sensitivity theory (Pluess & Belsky, 2013) suggests that some individuals are more sensitive to positive influences, such as therapy, so the observed effects may be driven by a subset of these ‘vantage sensitive’ individuals. This is an important consideration, especially given that it appears that different sensitivity subtypes may exist (Assary et al., 2021). Specifically, overall sensitivity appears to reflect a combination of genetic influences that each relate to positive and negative environmental sensitivities. As such, different configurations of these components in an individual may determine whether sensitivity manifests as a higher susceptibility to negative versus positive environmental influences. There are currently no measures that distinguish between these sensitivity subtypes, if they exist. Furthermore, little is known about the specific psychological processes that may facilitate a greater capacity for benefiting from interventions, an important question to be explored in future research.

We emphasise that research on sensitivity and treatment response is still in the early stages and requires further validation. We must take care not to conflate low sensitivity with low response to treatment. Rather, low‐sensitive individuals may respond equally well to a range of different treatment types, whereas more sensitive tend to be more discerning in their response, as found in Keers et al. (2016). Also, we would not expect sensitivity *alone* to inform clinical decision‐making about treatment, but if predictive of treatment outcome, then it could prove to be one useful factor for clinicians to consider, along with other demographic and clinical characteristics. Future research must take into consideration what works for the less sensitive, to ensure a balanced approach in the quest for ‘what works for whom’.

In the next section, we outline future research directions that can address these challenges and limitations of research to date.

## Future research directions for clinically relevant differential susceptibility research

Here, we propose several future research directions that would help move the potential clinical impact forward. These relate to creating better measures of sensitivity, determining its applicability to clinical practice and examining the mechanisms underlying the observed associations with clinical outcomes.

The currently available questionnaires are useful psychometrically validated tools for quantifying individual levels of environmental sensitivity, but have been criticised for being biased towards the negative aspects of sensitivity. Future research efforts in creating more balanced measures of sensitivity are essential if we are to understand the positive side of sensitivity. Such research would ideally include qualitative methods in the first step, in order to better understand the main features of both positive and negative sensitivities that underlie differential susceptibility to environments. Genetic measures of sensitivity need to be better powered, and thus able to account for a larger proportion of variance. Future studies thus require access to larger samples and using a larger range of phenotypes that include positive ones that relate to normal functioning.

Enhancing applicability of sensitivity to clinical practice requires more longitudinal prospective studies with clinical outcomes. For example, whilst highly sensitive personality scores in population‐based samples have been associated with higher depression symptom scores, these findings now need to be extended to clinical populations and diagnostic measures. This is important because it is possible (if unlikely) that sensitivity relates only to self‐reported depressive symptoms, but not to the development of major depressive disorder. On a related note, there are no studies identifying the cut‐off scores above which a sensitivity score may be associated with clinical psychopathology. Follow‐up research could also examine whether higher sensitivity, in interaction with stressors predicts disease onset or recurrences, in a prospective longitudinal design. This is an important line of research that would determine whether sensitivity is a significant clinical predictor of disorders, especially those disorders where significant life events play an important role (e.g. depression).

Finally, more work is needed to identify the mechanisms underlying sensitivity. Understanding these processes would aid the provision of more targeted interventions, focused on prevention and/or treatment of mental health problems. We believe there is a particular benefit in identifying factors that contribute to highly sensitive individuals' enhanced treatment outcomes. As alluded to earlier, this work needs to focus on both the positive as well as the negative end of the range when examining mechanisms. The findings from such research could be used to promote the psychological/cognitive strategies that sensitive individuals employ to benefit more from the positive aspects of their environment, and advance and maintain their well‐being. Of note, at present, little is known about how to maximise treatment response in individuals who are the least sensitive. Further research specifically with these individuals is essential for the provision of effective personalised treatments.

## Conclusions

Differential susceptibility theory and research have the potential to impact clinical practice in prevention and treatment of mental health disorders. However, at present, the theory and research findings cannot yet be used to inform direct clinical practice. Should the findings from current research be replicated and extended in future studies, environmental sensitivity could emerge as an important factor in identifying those who may be at higher risk of mental health problems in response to environmental risk factors. It could also offer an alternative model of psychopathology, that centres on an individual's sensitivity to the impact of positive, as well as negative experiences, rather than mere vulnerability. Research in this area has highlighted how treatment outcomes may differ as a function of an individual's environmental sensitivity. However, to capitalise on these potential clinical impacts, future research agendas would include studies to develop robust measures of sensitivity, and to examine the underlying mechanisms that increase the risk for mental health but also advantages in response to therapeutic interventions.

**Key points**

- Differential susceptibility theory suggests that individual differences in general sensitivity to one's context moderate response to both negative and positive environmental influences.
- Research findings indicate that higher sensitivity is associated with an increased risk of mental health problems in the context of adversity but also improved response to therapeutic interventions.
- Incorporating differential susceptibility thinking and research in clinical practice may impact the formulation and treatment of mental health problems, by considering general sensitivity to, rather than just vulnerability to, environmental exposures and by devising therapeutic plans that consider sensitivity as an important factor in treatment outcome.
- Further research should aim at understanding the biological and psychological mechanisms that render high sensitivity both risk and protective factor for mental health.
